# The chronnectome as a model for Charcot’s ‘dynamic lesion’ in functional movement disorders

**DOI:** 10.1016/j.nicl.2020.102381

**Published:** 2020-08-13

**Authors:** Ramesh S. Marapin, A.M. Madelein van der Stouwe, Bauke M. de Jong, Jeannette M. Gelauff, Victor M. Vergara, Vince D. Calhoun, Jelle R. Dalenberg, Yasmine E.M. Dreissen, Johannes H.T.M. Koelman, Marina A.J. Tijssen, Harm J. van der Horn

**Affiliations:** a Department of Neurology, University Medical Center Groningen, Hanzeplein 1, 9713 GZ Groningen, The Netherlands; b Expertise Center Movement Disorders Groningen, University Medical Center Groningen (UMCG), Groningen, The Netherlands; c Tri-institutional Center for Translational Research in Neuroimaging and Data Science (TReNDS), Georgia State, Georgia Tech, Emory, 55 Park Pl NE, Atlanta, GA 30303, United States; d Neurology and Clinical Neurophysiology, Amsterdam University Medical Center, location AMC, Amsterdam, The Netherlands

**Keywords:** Brain dynamics, Functional movement disorders, fMRI, Functional connectivity, Brain networks

## Abstract

This exploratory study set out to investigate dynamic functional connectivity (dFC) in patients with jerky and tremulous functional movement disorders (JT-FMD). The focus in this work is on dynamic brain states, which represent distinct dFC patterns that reoccur in time and across subjects. Resting-state fMRI data were collected from 17 patients with JT-FMD and 17 healthy controls (HC). Symptom severity was measured using the Clinical Global Impression-Severity scale. Depression and anxiety were measured using the Beck Depression Inventory (BDI) and Beck Anxiety Inventory (BAI), respectively. Independent component analysis was used to extract functional brain components. After computing dFC, dynamic brain states were determined for every subject using k-means clustering. Compared to HC, patients with JT-FMD spent more time in a state that was characterized predominantly by increasing medial prefrontal, and decreasing posterior midline connectivity over time. They also tended to visit this state more frequently. In addition, patients with JT-FMD transitioned significantly more often between different states compared to HC, and incorporated a state with decreasing medial prefrontal, and increasing posterior midline connectivity in their attractor, i.e., the cyclic patterns of state transitions. Altogether, this is the first study that demonstrates altered functional brain network dynamics in JT-FMD that may support concepts of increased self-reflective processes and impaired sense of agency as driving factors in FMD.

## Highlights

• We found altered functional brain state dynamics in FMD.
• Midline brain areas seem to play an important role in this process.
• Our findings may support theories about attention and sense of agency.
• Applying chronnectome framework in the future may discover novel biomarkers in FMD.

## 1 Introduction

Functional neurological disorders (FND) are commonly encountered neurological disorders, accounting for between 15 and 30% of neurology outpatients depending on how they are defined (Carson et al., 2000, Reid et al., 2002, Fink et al., 2005, Stone et al., 2010). Functional movement disorders (FMD), a subset of FND, are defined as genuine but incongruent with movement disorders that are regarded as “organic” in nature (Edwards and Bhatia, 2012). Patients with FMD suffer from weakness or involuntary movements resulting in increased disability and suffering (Hallett, 2006). The pathophysiological basis of FMD remains poorly understood. Given their high prevalence and significant impact on quality of life, further investigation into the neural correlates underlying FMD is warranted.

In recent years, the field of FMD has undergone a conceptual shift from abandoning an uniformly psychological etiology to incorporating neurobiological factors as key pathophysiological drivers (Czarnecki and Hallett, 2012). Importantly, in 2012, Edwards and colleagues proposed a neurobiological framework explaining functional motor and sensory symptoms from a perspective of pathological prior experiences that are modulated by attention dysregulation and altered sense of agency (Edwards et al., 2012). Patients with FMD have a body-focused attentional bias, which subsequently drives abnormal perceptions or movements. This role of attention is emphasized by the suppression of symptoms with distraction maneuvers, a core phenomenon in FMD (van der Stouwe et al., 2016). Sense of agency refers to the experience of controlling one's own actions and, through them, events in the external world (Haggard, 2017). This process is impaired in FMD, as patients report a lack of voluntary control over their movements (Edwards et al., 2013). Investigating the attentional process and sense of agency are therefore pertinent endeavors in patients with FMD.

Despite advances in neurobiological theories, FMD remain conceptually enigmatic, and it is thus critical to exploit new frameworks to better understand their underlying neural basis. Resting-state functional MRI (rsfMRI) is a non-invasive neuroimaging tool that can be used to investigate the functional organization of spatially separated brain regions, also known as functional brain networks (Biswal et al., 1995, Biswal et al., 2010). In this way, brain regions functionally implicated in e.g. motor tasks, could also be identified in rest. Previous fMRI studies have provided converging evidence that distinctive neural network aberrations are involved in the pathophysiology of FMD (Cojan et al., 2009, Voon et al., 2010, Nahab et al., 2011, van Beilen et al., 2011, Aybek et al., 2014, Maurer et al., 2016). Key aberrations have been found in areas associated with self-monitoring and attention such as the precuneus and posterior cingulate cortex (PCC), and in areas associated with the sense of agency such as the temporoparietal junction (TPJ). In addition to investigating etiological mechanisms, rsfMRI has potential for monitoring treatment responses in FMD. For example, Espay and colleagues have shown that clinical improvement is linked to changes in the anterior cingulate after cognitive behavior therapy, using task-based fMRI in patients with functional tremor (Espay et al., 2019).

So far, functional connectivity studies in FMD have used static functional connectivity analyses, which are applied under the assumption that connectivity patterns of spatially separated brain regions remain constant over time. However, human minds are not static but rather constantly switch between thoughts and cognitive states, and naturally, the connectivity patterns between brain regions fluctuate accordingly (Hutchison et al., 2013, Allen et al., 2014, Calhoun et al., 2014). These fluctuations in functional connectivity are referred to as dynamic functional connectivity (dFC), and the entire network of dynamic connections throughout the human brain as the human ‘chronnectome’ (Chang and Glover, 2010, Calhoun et al., 2014, van der Horn et al., 2019).

Interestingly, already in 1899, Jean-Martin Charcot regarded FMD as a manifestation of a ‘dynamic lesion’ which escapes our ‘present means of investigation’ (Aybek, 2019). In this regard, the advent of the chronnectome-framework as a novel investigational tool can be pivotal in more aptly capturing and understanding the brain changes underlying the ‘dynamic lesion’ in FMD (Hutchison et al., 2013, Calhoun et al., 2014, Lurie et al., 2020). Recently, Diez and colleagues used a novel analysis technique called stepwise functional connectivity, which characterizes the propagation and convergence of functional connectivity across brain networks (Sepulcre et al., 2012, Diez et al., 2019). Using stepwise functional connectivity, the authors describe candidate neurocircuit pathways in the pathophysiology of FND, thus expanding upon current conceptualizations surrounding the underlying nature of FMD. It is important to note, however, that this method is still based on static functional connectivity.

Similarly exploring new avenues of study, we endeavored to assess fluctuations in functional connectivity by means of dFC in patients with FMD. Our focus was on dynamic brain states, which are time-related patterns of dFC identified with a data-driven clustering approach. Put simply, these patterns can be thought of as prototypical functional connectivity patterns that subjects tend to return to across time (Calhoun et al., 2014). Similar to other neurologic diseases, such as traumatic brain injury and stroke, it is plausible that changes in the dynamics of brain states are related to the etiology of FMD (Bonkhoff et al., 2019, van der Horn et al., 2019). Furthermore, it has been shown that in some cases different brain states transition between one another via cyclic patterns of connectivity increases and decreases (van der Horn et al., 2019). These cyclic patterns of brain transitions are called *attractors*, which can be disrupted due to brain disorders, such as traumatic brain injury (van der Horn et al., 2019, Vergara et al., 2019b). The primary aim of our exploratory resting-state fMRI study is to investigate whether the properties and behavior of dynamic brain states differ between FMD patients and healthy controls (HC). We further investigate whether characteristics of brain states are related to self-reported symptom severity, anxiety, and depression in FMD patients. To the best of our knowledge, the current study is the first to investigate resting-state dFC in patients with FMD.

## 2 Materials and methods

### 2.1 Participants

Seventeen patients with a clinical diagnosis of jerky and/or tremulous FMD (JT-FMD), comprised of functional tremor and/or functional jerks, were included. This sample size was chosen for feasibility reasons. Patients were recruited from the movement disorder clinics of the University Medical Center Groningen and the Amsterdam University Medical Center, location AMC, the Netherlands (Dreissen et al., 2019). Seventeen age-, sex-, and education-matched HC were recruited via poster ads in the community and the internet. The diagnosis of functional tremor or myoclonus was confirmed by two movement disorder experts (M.T. & J.H.T.M.K.) in their respective clinics according to the current DSM-5 criteria and by using positive findings in the history and neurological examination. Exclusion criteria were: age <18 years; comorbid neurological disorder; contraindications for MRI-scanning; patients with disruptive jerky movements of the head, as this could lead to spurious results due to head motion artefacts in the data (Power et al., 2012); and patients using antipsychotic drugs, as antipsychotic medications are known to affect brain activation (Lui et al., 2010). One patient was using benzodiazepines at the time and was asked to discontinue their medication one day prior to the scan. All participants in the study provided written informed consent. The study was approved by the medical ethics committee of the Amsterdam University Medical Center, location AMC, the Netherlands. Study procedures were conducted according to the declaration of Helsinki. Participants were scanned between January 2014 and November 2016. Patients were included in a broader study (trial registration number: NTR2478), these data will be published elsewhere to maintain transparency for the presented results here.

### 2.2 Clinical assessment

Patients with JT-FMD self-rated symptom severity using the Clinical Global Impression Severity (CGI-S) Scale, a 7-point Likert-item ranging from 1 to 7 (1 = normal, I have no complaints, 7 = severe). Patients also completed the Beck Depression Inventory (BDI) and Beck Anxiety Inventory (BAI; Beck et al., 1988, Beck et al., 1961). HC did not undergo psychometric testing.

### 2.3 Magnetic resonance imaging acquisition

Functional and structural imaging data were acquired with a 3.0 Tesla MRI scanner (Philips Intera Medical Systems, Best, the Netherlands) using a 32-channel SENSE head coil. Participants lay head-first supine in the scanner. An axial T1-weighted 3D turbo field echo (T1TFE) sequence image was acquired for anatomical reference: TR 9 ms; TE 3.5 ms; number of echoes 1; flip angle 8°; matrix size = 256 × 256; FOV: 232 × 170 × 256 mm; voxel size 1 × 1 × 1 mm; acquisition time: 4 min 18 s. With respect to functional imaging, 225 T2-weighted fast field single echo with echo planar imaging (FEEPI) sequence volumes were acquired, each with 39 slices aligned in the anterior commissure-posterior commissure plane and recorded in descending order: repetition time (TR) 2,000 ms; echo time (TE) 30 ms; flip angle 70°; matrix size = 64 × 62; field of view 224 × 137 × 224 mm; voxel size 3.5 mm × 3.5 mm × 3.5 mm; acquisition time: 7 min and 30 s. One run was collected per participant. All imaging data was acquired in one session. During the rsfMRI scan, patients were instructed to remain as still as possible, including suppression of involuntary movements, to keep their eyes open and look in front of them, to remain awake and to think of nothing.

### 2.4 Preprocessing

Functional images were preprocessed using SPM12 (Wellcome Department, University College London, England) in Matlab version R2019b (MathWorks, Natick, MA, USA). Images were realigned, coregistered with the anatomical scans, and normalized to MNI-space using an EPI-template. Prior to smoothing, voxel time courses were orthogonalized with respect to the six realignment parameters and their first order derivatives, as well as to linear, quadratic, and cubic trends (Vergara et al., 2017a, Vergara et al., 2017b). Framewise displacement (FD) was estimated for each group, calculated as the sum of the absolute values of the derivatives of the six realignment parameters generated in the preprocessing step (Power et al., 2012). There were no differences in mean FD between groups (t = 0.2294; p = 0.82; see Supplementary Fig. 1). The first three volumes were discarded to account for T1-disequilibrium effects. An 8 mm full width at half maximum Gaussian kernel was used for smoothing.

### 2.5 Independent component analysis (ICA)

Group-ICA using a multi-objective optimization spatially constrained ICA (scICA) in the Group ICA of fMRI toolbox (GIFT; version 4.0b) was used to compute brain components for dFC analysis (Calhoun et al., 2001, Du and Fan, 2013). Fifty-three components from the NeuroMark framework were used as templates for scICA (Du et al., 2019), resulting in 53 subject-specific components (each consisting of a spatial map, and a time course) per person. These component time courses were then used to compute connectivity matrices. This set of neural components was grouped into the following seven functional subdomains: auditory (AUD; 2 components), cerebellar (CER; 4 components), cognitive control (CON; 17 components), default mode network (DMN; 7 components), subcortical (SCN; 5 components), sensorimotor (SMN; 9 components), visual (VIS; 9 components).

### 2.6 Dynamic functional connectivity analyses

For a detailed description of the dFC analyses we kindly refer the reader to our earlier work (van der Horn et al., 2019). Below follows a brief summary of the analyses. The processing pipeline used in this study is shown in Supplementary Fig. 2. For every participant, dFC was computed on the components’ time courses using GIFT functions (yielding 200 windows per subject, with each window containing 53 × 52/2 = 1378 Pearson correlations; window size: 22 TR = 44 s). Prior to computing dFC, component time courses were despiked and filtered using a fifth-order Butterworth bandpass filter (0.01–0.15 Hz). Successive windows were shifted in steps of 1 TR each, which means that windows are overlapping. Subsequently, average sliding window correlations (ASWC; window size = 25 TR) and the first order derivatives were calculated for every subject, resulting in 175 windows per subject, with each window containing 1378 × 2 = 2756 values (Espinoza et al., 2019, Vergara et al., 2019a). We opted for the ASWC approach, as this requires smaller window lengths, and thus a more accurate estimation of dFC, by reducing spurious fluctuations, as compared to the standard sliding window correlation approach (Vergara et al., 2019a). The first-order derivatives encompass the connectivity changes between two contiguous windows (‘speed’ of connectivity change; Calhoun et al., 2014, Espinoza et al., 2019). We included these first order derivatives in our analysis as it has been shown that this facilitates the estimation of the optimal number of dynamic brain states, and thus results in a higher sensitivity of finding neuropsychiatric disease-related patterns (Espinoza et al., 2019).

To find the optimal number of clusters (k), the Davies-Bouldin and Ray-Turi cluster validity indices were computed for a range of k (2–10) using subject-specific local maxima in variance of the ASWC and derivatives (i.e., windows showing the highest variance), resulting in an optimal k = 4 (Vergara et al., 2020). Here, clusters represents groups of connectivity matrices (i.e., the data points) with similar patterns of ASWC and derivatives. Supplementary Fig. 3 shows a 2D representation of the four dynamic brain states. The optimal number of k was also estimated using either the total ASWC or derivatives, which both resulted in k = 5. Supplementary Document 1 shows all of the results for k = 5, which are similar to those with k = 4 in the main manuscript.

For every subject, the ASWC and derivatives were Z-scored separately before running k-means clustering (also with regard to cluster estimation). All windows across all subjects were then concatenated (in a (175*34) × 2756 size matrix) and clustered (Distance Measure = Correlation, Maximum Iterations = 4000, number of replicates = 33), resulting in the assignment of a state index (1 to 4) to every window of every subject. State indices were used to calculate the following state-clustering measures: mean dwell time (MDT), fraction of time spent in every state (FT), number of state transitions, and number of visits per state. Regarding the number of transitions, also the number of specific transitions between states (six unique combinations in two directions = 12 possible transitions) were computed. State transitions were examined more closely by focusing on the probability of transitioning from one state to the other. Previous research on this topic has shown that transitioning between dynamic functional brain states in healthy subjects occurs in oscillatory patterns that orbit a center of functional connectivity, patterns which are named *attractors* (van der Horn et al., 2019, Vergara et al., 2019b). We explored differences in attractors between HC and patients with JT-FMD.

### 2.7 Statistical analyses

For clinical measures, analyses were performed using the Statistical Product and Service Solutions (SPSS, version 25, IBM Corp., Armonk, NY). The normality of distribution of continuous variables was tested by one-sample Kolmogorov-Smirnov test. Continuous variables with normal distribution were presented as mean (standard deviation [SD]); non-normal variables were reported as median (interquartile range [IQR]). For demographics and clinical characteristics, student’s *t* tests were used to test for any between-group differences of continuous data. For nominal data, Chi-square tests were performed. Alpha was set at 0.05 (two-tailed). Furthermore, we ensured groups were age-, gender-, and education-matched. Group differences in state-clustering measures were examined using permutation tests in Matlab (two-tailed, α = 0.05, 100,000 permutations). For state-related measures (MDT and FT), false discovery rate (FDR) corrections were applied for multiple testing (m = 4; Benjamini and Hochberg, 1995). Hedges’ *g* effect sizes were computed using the Measures of Effect Size Toolbox in Matlab (Hentschke and Stüttgen, 2011). Analyses of state-transition probabilities rely on data of the whole group (HC or JT-FMD), and therefore there is no subject-wise information that can be used for statistical analyses. In addition, for the patient group Spearman correlations were computed between CGI, BAI, and BDI scores on the one hand and dynamic measures on the other (α = 0.05 with FDR corrections in case of multiple testing).

## 3 Results

### 3.1 Demographics and clinical characteristics

Data from 17 patients with JT-FMD and 17 age-, sex-, and education-matched HC were included in the analysis. Patient demographic and clinical characteristics are summarized in Table 1. There were no differences in age, sex and education level between the patient and control groups, which was expected as the groups were matched on these parameters (P > 0.05). We found no clinically significant depression (median BDI = 7, IQR = 3.5) or anxiety (median BAI = 13, IQR 23) in the JT-FMD patients.

**Table 1:** Patient characteristics.

	JT-FMD patients (n = 17)	Healthy controls (n = 17)	P-value
Age, mean (SD), years	43.6 (14.4)	43.2 (14.5)	0.93
Sex, females/males	9/8	9/8	1.00
Education level, less than higher professional education	11	7	0.17
Predominant type of symptom, jerks/tremor	4/13	NA	NA
Disease duration, mean (SD) years	4.1 (3.0)	NA	NA
Symptom location, extremities/axial	13/4	NA	NA
CGI-S Score (0–7), median (IQR)	5 (2)	NA	NA
BDI Score (0–63), median (IQR); range	7 (3.5); range 0–28	NA	NA
BAI Score (0–63), median (IQR); range	13 (23); range 0–38	NA	NA

Abbreviations: JT-FMD = jerky and tremulous functional movement disorders; CGI-S = clinical global impression-severity scale; NA = not applicable; data are presented as the mean ± SD unless specified otherwise.

### 3.2 Dynamic brain states

**Fig. 1:**
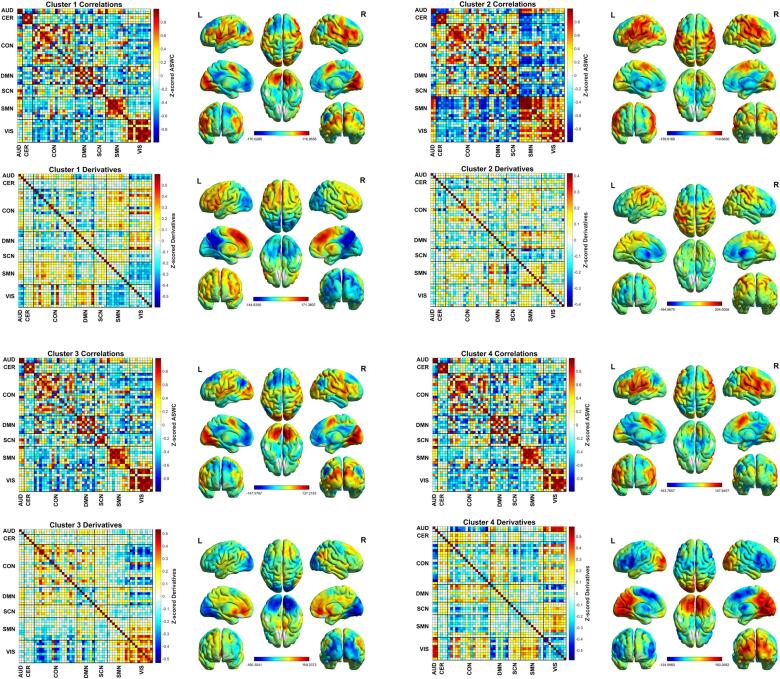
Centroids (i.e., mean dFC or derivatives which form the center point of a cluster) for every state calculated for the entire study sample. The majority of all windows was assigned to state 1 (33%), followed by state 3 (30%), state 4 (25%), and state 2 (12%). Rendered brain images depict the components’ t-maps weighted by the sum of Z-scores for ASWC and derivatives for every component. Functional connections were rendered on brain surfaces using BrainNet viewer (Xia et al., 2013). ASWC = average sliding window correlations; AUD = auditory; CER = cerebellar; CON = cognitive control; DMN = default mode network; SCN = subcortical; SMN = sensorimotor; VIS = visual.

Prototypical functional connectivity patterns for the four states are depicted in Fig. 1. As can be seen, ASWC for state 1, 3, and 4 are fairly similar. State 1, 3, and 4 in general show high connectivity within subdomains, with mostly negative correlations between subdomains. State 2 was visited by six of the HC, and three of the JT-FMD group. State 2 is characterized by positive correlations between CER, CON, DMN, and SCN on the one hand, and between SMN and VIS on the other. Between these two subdomains of components there were mainly negative correlations. Differences between states 1, 3, and 4 are predominantly based on the first order derivatives of the ASWC. State 1 shows increasing functional connectivity (positive derivatives) of components within the medial prefrontal cortex (MPFC), and to a lesser extent in the lateral prefrontal cortices, together with decreasing posterior midline connectivity. State 4 shows the opposite pattern. Furthermore, when inspecting the correlation matrices, there is increasing and decreasing connectivity between DMN and VIS, and between CON and SMN for state 1 and 4, respectively. For DMN with SMN and CON, the opposite seems to be true. Both state 1 and 4 show decreasing connectivity of components within the posterior midline. State 3 shows strongly decreasing connectivity within the occipital areas, however, there are less clearly defined derivatives for the frontoparietal midline areas compared to state 1 and 4. The correlation matrix shows a less segregated derivative pattern for state 2.

### 3.3 State clustering analyses

**Fig. 2:**
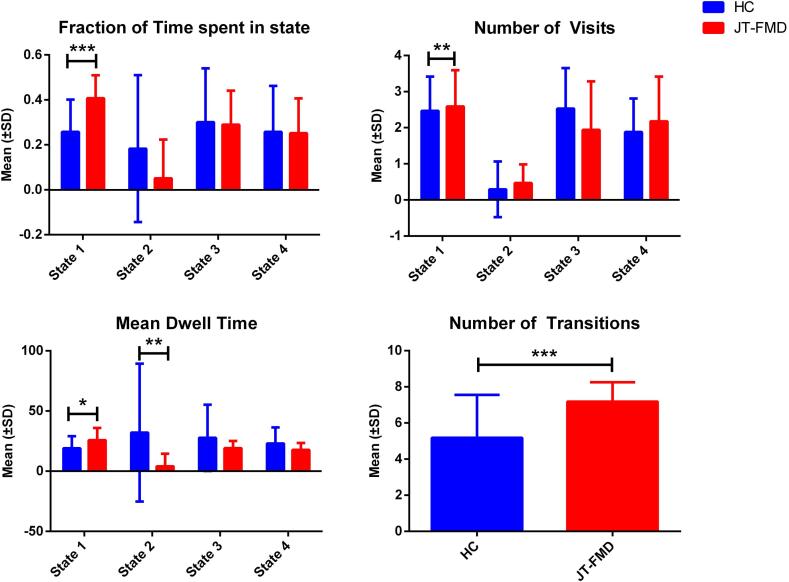
Group differences for state-clustering measures. *** Significant after at *P* < 0.05 with FDR correction, ** *P*_uncorrected_ < 0.05, * *P* < 0.1.

In Fig. 2, the fraction of time spent in every state for HC and JT-FMD is depicted. It can be appreciated from this figure that state 2 is the least frequently visited state for both groups. Patients with JT-FMD spent significantly more time in state 1 compared to HC (*P* = 0.0014, *g* = 1.17; Fig. 2). There was also a trend toward visiting this state more often (*P* = 0.0469, *g* = 0.76). Regarding dwell time there was a similar, but not statistically significant effect visible for state 1 (*P* = 0.0575, *g* = 0.65). In addition, patients showed a trend toward dwelling less in state 2 (*P* = 0.0383, *g* = -0.67). Furthermore, patients with JT-FMD showed a higher number of transitions than HC (*P* = 0.0033, *g* = 1.06).

### 3.4 State transitions

**Fig. 3:**
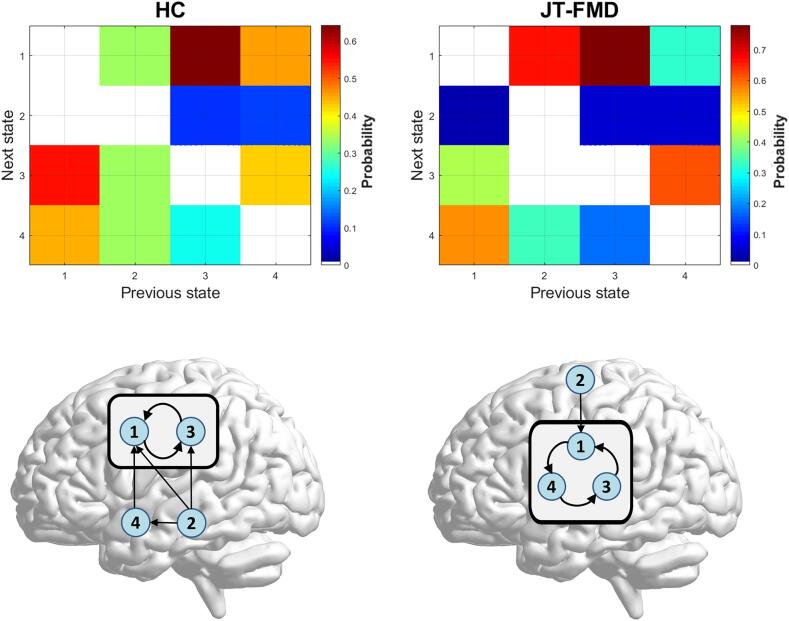
Attractor and transition probabilities for HC and JT-FMD. Top images show transition probabilities. Bottom images show a schematic representation of state transitions with the highest probability (i.e., attractor).

States that transition with the highest probability into other states form *attractors*, which represent cyclic patterns of functional connectivity around a center (Fig. 3). In HC, the attractor is made up by state 1 and 3, whereas in JT-FMD patients the attractor is composed of three states (1, 3 and 4). In other words, state 4 is now incorporated in the attractor. For HC, state 2 transitions with the highest probability into all other states; for JT-FMD it transitions into state 1. The Supplementary material section contains a movie showing state transitions for a patient with JT-FMD.

**Fig. 4:**
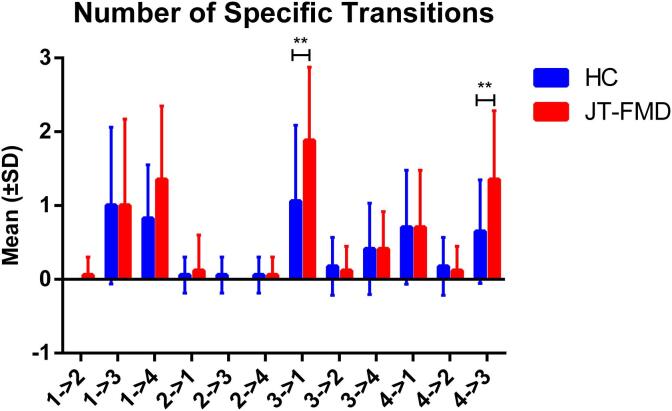
Specific state transitions. ** *P*_uncorrected_ < 0.05.

Regarding specific transitions, trends were observed toward more frequent transitions from state 3 to state 1 (*P* = 0.0379, *g* = 0.8; Fig. 4), and from state 4 to state 3 in patients with JT-FMD (*P* = 0.0303, *g* = 0.84).

### 3.5 Associations between dynamic measures and self-rated symptom severity, anxiety, and depression

No significant correlations were found between these measures.

## 4 Discussion

Despite recent advances in neurobiological theories, our understanding of the mechanisms that drive FMD remains insufficient. Thus far, functional imaging studies conducted on FMD samples have focused on static functional connectivity and essentially ignore the dynamics of mental states underlying FMD. This exploratory study set out to gain further insight in neuronal mechanisms involved in the pathophysiology of FMD by investigating the functional chronnectome in patients with JT-FMD using resting-state fMRI. The present study is the first to report on resting-state dynamic functional connectivity in patients with FMD. In summary, patients with JT-FMD spent significantly more time in a state characterized by increasing medial prefrontal and decreasing posterior midline connectivity. Patients also more frequently visited this state. In addition, patients transitioned more often between dynamic brain states, exhibiting higher dynamism on multiple measures. Finally, the attractor profiles differed between groups, with patients with JT-FMD exhibiting a cyclic pattern of transitioning between three instead of two states, in which they more strongly incorporate a state with decreasing medial prefrontal and increasing posterior midline connectivity.

The observation that patients with JT-FMD spent more time in a state with incrementing connectivity in the medial prefrontal cortex, an important area of the default mode network, could reflect a state of heightened self-monitoring and self-referential processing (Amodio and Frith, 2006, Northoff et al., 2006, Mason et al., 2007, Cojan et al., 2009, Vuilleumier, 2014, Raichle, 2015). These results align with and corroborate findings from earlier studies in FMD, where the medial prefrontal cortex was found to be aberrantly active (Cojan et al., 2009, de Lange et al., 2007, Hassa et al., 2016, Voon et al., 2010). Also in accordance with our findings, Kaiser and colleagues provided evidence that depression and ruminative thinking in depression are related to abnormal patterns of dynamic functional connectivity in the medial prefrontal cortex (Kaiser et al., 2016). However, it should be noted that the method used in their study is different from the one used in the current paper, and we did not find any significant correlations between fraction of time spent and depression scores. The finding that patients changed states more often compared to healthy controls, and exhibited an attractor profile which additionally incorporated a state with decreasing frontal and increasing parietal connectivity (Fig. 3), could also indicate a possible compensatory mechanism to overcome excessive introspective processes necessary to breach ruminative and undermining attentional processes. Thus, these observations may be interpreted as manifestations of altered self-referential processes, which are either causative in or in response to the development of FMD.

We acknowledge that this postulation remains to have a tentative character. E.g., as patients were not debriefed after scanning, one might oppose that these findings could also be due to the fact that patients were perhaps more vigilant and thus more easily distracted compared to the healthy controls, ultimately driving the observed differences. Another important point to consider is that abnormal medial prefrontal cortex activity and aberrant functional connectivity have been demonstrated in many neuropsychiatric and neurodegenerative disorders. While we relate the well-recognized contribution of the medial prefrontal cortex (DMN) to self-referential processes and ruminative thinking, the possible involvement of vigilance and rumination lacks specificity. A more specific understanding of this relationship in FMD would require a comparison to control subjects matched in terms of depressive symptoms and to depressed subjects without a functional movement disorder. Finally, regarding the state with increasing medial prefrontal connectivity, increasing connectivity was also observed between the sensorimotor and cognitive domains (e.g. insula, cingulate gyrus), which is in line with the study of Diez and colleagues (Diez et al., 2019). In FMD, compared to HC, they found increased first link-step functional connectivity from primary motor areas to medial motor-related regions such as the SMA and middle cingulate cortex. Furthermore, they found increased second link-step functional connectivity to additional multimodal integration areas such as the insula and temporo-parietal junction, while more distant dorsomedial prefrontal cortex showed stronger third link-step interconnection with the motor cortex in patients. This pattern of increased functional connectivity was regarded to represent aberrant integration of sensorimotor, interoceptive, attentional, and emotional information (Diez et al., 2019).

Our results are consistent with concepts concerning changes in internal monitoring of action and perception underlying FMD. We propose that spending excessive time in a brain state characterized by increasing (dorso)medial prefrontal connectivity, as demonstrated in the current study, might point toward altered cognitive processes involved in the predictions and perceptions of movement consequences. From a Bayesian perspective, Edwards and colleagues hypothesized that functional motor symptoms are caused and sustained by an imbalance between bottom-up sensory evidence and top-down sensory predictions leading to a distorted perception of the proprioceptive consequences of movements (Edwards et al., 2012). Cortical midline structures play an important role in this process, which they illustrate with an example of motor control, centered around the SMA and pre-SMA. At this level of motor control, proprioceptive predictions are pre-eminent. Taken together with the above described findings of Diez et al. (2019), enhanced functional interconnection with the dorsomedial prefrontal cortex bordering the (pre-)SMA, the DMN core region implicated in our study, may well point at the contribution of attentional predictions, i.e., higher-order motor predictions concerning purposeful movement going beyond basic sensorimotor predictions (see also Baizabal-Carvallo et al., 2019). We theorize that increased state transition frequency found in our study may indicate an augmentation of information flow in the dynamic neural system in an attempt to adequately align predictions and perceptions. These predictions are then relayed in a top-down direction to test if they match the initiated effector movements. Predictive neural representations of behavior have also been referred to as ‘efference copies’, or ‘corollary discharges’, which are forwarded to the sensory system to be matched with the sensory feedback caused by the movements (‘self-generated reafference’; Straka et al., 2018). In addition to the prefrontal cortex, the parietal cortex and cerebellum play an important role with regards to this process (de Jong, 2019). According to Edwards *et al.’s* model, in FMD, abnormal prior beliefs about movements are generated by areas that occupy intermediate steps in the motor hierarchy, such as the SMA, and are given excessive precision (weight) due to heightened attentional processes. Subsequently, these abnormal beliefs override the influence of bottom-up sensory evidence and give rise to prediction errors, which subsequently induce autonomous reflex-like movements at the lower (effector) levels in the motor hierarchy. At the same time, these errors are conveyed to higher order prefrontal areas which in turn explain them as being unintended, in order to minimize the amount of free energy or surprise in the total system (i.e., the difference between prediction and perception of movement and attended action; Friston et al., 2006). Thus, according to this model, the misinterpretation of bottom-up generated prediction errors by higher order areas causes an abnormal sense of agency and leads to the perception of ‘symptoms’. The observation in the current study that patients are more likely to transition between states with either increasing or decreasing prefrontal connectivity might point towards changes in higher order prefrontal processes underlying the misinterpretation of prediction errors as symptoms in which attention regulation plays an integrative role. In a complementary fashion, this prefrontal involvement could be a manifestation of synaptic gain related to increased attention to prior beliefs. The state that was incorporated more strongly in the attractor in patients with JT-FMD, also shows increasing connectivity of the precuneus, an area that is indeed important for attention shifting. Thus, it could be hypothesized that patients try to compensate for attention distortions by engaging this state. Taken together, our results point toward changes in the dynamic neural architecture underlying pathological afferent and efferent higher-order motor processes in FMD. Because subjects were instructed to suppress their movements, and given the role of the SMA in the initiation and suppression of movements, we compared overall (static) functional connectivity among the sensorimotor components between patients and controls (Supplementary Fig. 4; Potgieser et al., 2014). No significant differences were found, and therefore we believe this instruction did not influence our results to a large extent. We do, however, acknowledge the tentative character of our explanations. Future task-fMRI studies that make use of dynamic functional connectivity analyses might shed further light on the matter.

There are several strengths of our study. First, the functional chronnectome was investigated not only by focusing on component functional connectivity at certain points in time, but also from a first-order-derivative-perspective, which encompasses the changes of connectivity between two consecutive windows (‘speed’ of connectivity change; (Calhoun et al., 2014, Espinoza et al., 2019). It has been shown that including these first order derivatives facilitates the estimation of the optimal number of dynamic brain states, and ultimately results in a higher sensitivity of finding neuropsychiatric disease-related patterns (Espinoza et al., 2019). Second, by including derivatives we demonstrated that dynamic brain states seem to adhere to specific transition sequences, called attractors, with dynamic connectivity increasing and decreasing in orbiting patterns. In the present study we examined differences in attractors between patients with JT-FMD and healthy controls. Third, we implemented a fully automated spatially constrained ICA (GIG-ICA) method, which has been shown to yield better performance than existing techniques (such as single subject ICA) with respect to artifact removal and spatial and temporal accuracy, providing more reliable functional components (Du and Fan, 2013, Du et al., 2016). We used a set of independent template components obtained from almost 2000 healthy controls (Du et al., 2019), which circumvents any possible bias associated with estimation of functional subunits using the data itself. Finally, the average sliding window approach reduces spurious fluctuations, which allowed us to choose a smaller window size compared to a sliding window correlation approach (Vergara et al., 2019a).

We acknowledge that our study also has limitations. First, there was a relatively small sample size (34 participants), which could have resulted in false negative findings. However, the use of permutation statistics gives us confidence in the reported group differences. It also has to be mentioned that in the current literature, most studies conducted in patients with FMD consist of sample sizes smaller than 30 patients. As there seems to be overlap across the FMD spectrum our findings are likely to be generalizable, however, this is not a foregone conclusion and this phenotypic heterogeneity (functional tremor and functional myoclonus) needs to be acknowledged as a potential limitation. Another limitation is that patients’ clinical presentations are dynamic as well, with varying manifestations, severities, durations, and so on, while we used singly measured severity scores for our analysis (CGI-S, BDI, and BAI). Furthermore, we know that other factors, such as inattention, can also differ between FMD patients and HC and might thus influence the results. However, currently this is the best method available. A recent paper from the FND field acknowledges the lack of good outcome measures for FND patients (Nicholson et al., 2020). Here, we considered the CGI-S scale the best score, as it reflects the patients’ subjective severity taking into account more than just the motor score. Finally, the absence of psychometric measures in healthy controls prevents us from drawing hard conclusions on psychometric correlations.

## 5 Conclusion

In recent years, there has been a departure from an unwavering psychological etiology towards neurobiological factors driving FMD. Our study demonstrates altered brain network dynamics in JT-FMD, that may support concepts of increased self-reflective processes and impaired sense of agency as driving factors in FMD. Importantly, the dFC approach we used may help find key biomarkers idiosyncratic to FMD. Future studies should exploit the chronnectome framework across the entire FMD spectrum to further extend and consolidate the evidence garnered in this study.

## CRediT authorship contribution statement

**Ramesh S. Marapin:** Conceptualization, Formal analysis, Data curation, Writing - original draft. **A.M. Madelein van der Stouwe:** Writing - review & editing, Supervision. **Bauke M. de Jong:** Writing - review & editing, Supervision. **Jeannette M. Gelauff:** Methodology, Investigation, Writing - original draft, Writing - review & editing, Supervision, Project administration, Funding acquisition. **Victor M. Vergara:** Writing - review & editing. **Vince D. Calhoun:** Writing - review & editing. **Jelle R. Dalenberg:** Writing - review & editing. **Yasmine E.M. Dreissen:** Conceptualization, Methodology, Investigation, Project administration. **Johannes H.T.M. Koelman:** Writing - review & editing. **Marina A.J. Tijssen:** Conceptualization, Writing - review & editing, Funding acquisition. **Harm J. van der Horn:** Conceptualization, Formal analysis, Software, Data curation, Writing - original draft, Writing - review & editing.

## Declaration of Competing Interest

The authors declare that they have no known competing financial interests or personal relationships that could have appeared to influence the work reported in this paper.

## Appendix A Supplementary data

Supplementary video 1 In this movie the state transitions for one patient with JT-FMD are visualized. The top left animated plot (made using Matlab R2017b) demonstrates the change in state membership indices from window to window. In the right-hand figures, the time-related changes in average sliding window correlations and 1st order derivatives are depicted, with red and blue meaning positive (for correlations) / increasing (for derivatives) and negative/decreasing values, respectively. For every window, the sum of all 52 correlations and derivatives was computed for every component, with subsequent variance normalization. These values were then used to weigh the components’ Z-maps obtained with ICA. The weighted Z-maps were rendered on brain surfaces using BrainNet viewer (Xia et al., 2013), and all renderings across windows were combined in a movie using Matlab.

Supplementary figure 1 Mean framewise displacement for JT-FMD and HC . Lines reflect group mean ± standard deviation. There were no differences between groups (t=0.2294; p=0.82), which means that any movements present by extremities did not translate into head motion.

Supplementary figure 2 Dynamic functional network analytical pipeline

Supplementary figure 3 2D representation of the four dynamic brain states extracted from this dataset. A principal component analysis was run on all high dimensional windowed data (i.e., 1378 ASWC + 1378 derivatives (variance scaled to ASWC)). The scores for the first two principal components (PC) were then smoothed, interpolated, and visualized in a bivariate histogram plot for each state separately using the membership indices obtained from k-means clustering (all steps were conducted using Matlab). Distinct density peaks, which represent the frequency of occurrence of a certain x (PC2) - y (PC1) score in the 2D histogram, can be appreciated for the different states, which support the structure of the clustered data. It can be noticed that density peaks of state 1 and 3 are relatively close to each other, which matches the observation that these states form an attractor in healthy subjects, as will be explained in more detail later in the manuscript. ASWC = average sliding window correlations. .

Supplementary figure 4 Static functional connectivity among the sensorimotor components between JT-FMD and HC.

## References

[b0005] Allen, E.A., Damaraju, E., Plis, S.M., Erhardt, E.B., Eichele, T., Calhoun, V.D., 2014. Tracking whole-brain connectivity dynamics in the resting state. Cereb Cortex 24, 663–676. 10.1093/cercor/bhs352 PMC3920766 23146964

[b0010] Amodio, D.M., Frith, C.D., 2006. Meeting of minds: The medial frontal cortex and social cognition. Nat. Rev. Neurosci. 7, 268–277. 10.1038/nrn1884 16552413

[b0015] Aybek S. (2019). Corticolimbic fast-tracking in functional neurological disorders: towards understanding of the ‘dynamic lesion’ of Jean-Martin Charcot. J. Neurol. Neurosurg. Psychiatry.

[b0020] Aybek S., Nicholson T.R., Zelaya F., O’Daly O.G., Craig T.J., David A.S., Kanaan R.A. (2014). Neural correlates of recall of life events in conversion disorder. JAMA Psychiatry.

[b0025] Baizabal-Carvallo J.F., Hallett M., Jankovic J. (2019). Pathogenesis and pathophysiology of functional (psychogenic) movement disorders. Neurobiol. Disease.

[b0030] Beck A.T., Epstein N., Brown G., Steer R.A. (1988). An inventory for measuring clinical anxiety: psychometric properties.. J. Consult. Clin. Psychol..

[b0035] Beck A.T., Ward C.H., Mendelson M., Mock J., Erbaugh J. (1961). An inventory for measuring depression. Arch. Gen. Psychiatry.

[b0040] van Beilen, M., de Jong, B.M., Gieteling, E.W., Renken, R., Leenders, K.L., 2011. Abnormal parietal function in conversion paresis. PLoS One 6, 25918. 10.1371/journal.pone.0025918 PMC3200327 22039428

[b0045] Benjamini Y., Hochberg Y. (1995). Controlling the false discovery rate: a practical and powerful approach to multiple testing. J. Roy. Stat. Soc.: Ser. B (Methodol.).

[b0050] Biswal B., Zerrin Yetkin F., Haughton V.M., Hyde J.S. (1995). Functional connectivity in the motor cortex of resting human brain using echo-planar mri. Magn. Reson. Med..

[b0055] Biswal B.B., Mennes M., Zuo X.-N., Gohel S., Kelly C., Smith S.M., Beckmann C.F., Adelstein J.S., Buckner R.L., Colcombe S., Dogonowski A.-M., Ernst M., Fair D., Hampson M., Hoptman M.J., Hyde J.S., Kiviniemi V.J., Kotter R., Li S.-J., Lin C.-P., Lowe M.J., Mackay C., Madden D.J., Madsen K.H., Margulies D.S., Mayberg H.S., McMahon K., Monk C.S., Mostofsky S.H., Nagel B.J., Pekar J.J., Peltier S.J., Petersen S.E., Riedl V., Rombouts S.A.R.B., Rypma B., Schlaggar B.L., Schmidt S., Seidler R.D., Siegle G.J., Sorg C., Teng G.-J., Veijola J., Villringer A., Walter M., Wang L., Weng X.-C., Whitfield-Gabrieli S., Williamson P., Windischberger C., Zang Y.-F., Zhang H.-Y., Castellanos F.X., Milham M.P. (2010). Toward discovery science of human brain function. Proc. Natl. Acad. Sci..

[b0060] Bonkhoff, A.K., Espinoza, F.A., Gazula, H., Vergara, V.M., Hensel, L., Michely, J., et al., 2019. Acute ischemic stroke alters the brain’s preference for distinct dynamic connectivity states. medRxiv, 19011031. 10.1093/brain/awaa101 PMC7241954 32357220

[b0065] Calhoun V.D., Adali T., Pearlson G.D., Pekar J.J. (2001). A method for making group inferences from functional MRI data using independent component analysis. Hum. Brain Mapp..

[b0070] Calhoun V., Miller R., Pearlson G., Adalı T. (2014). The chronnectome: time-varying connectivity networks as the next frontier in fMRI data discovery. Neuron.

[b0075] Carson A.J., Ringbauer B., Stone J., McKenzie L., Warlow C., Sharpe M. (2000). Do medically unexplained symptoms matter? A prospective cohort study of 300 new referrals to neurology outpatient clinics. J. Neurol. Neurosurg. Psychiatry.

[b0080] Chang C., Glover G.H. (2010). Time–frequency dynamics of resting-state brain connectivity measured with fMRI. NeuroImage.

[b0085] Cojan Y., Waber L., Carruzzo A., Vuilleumier P. (2009). Motor inhibition in hysterical conversion paralysis. NeuroImage.

[b0090] Czarnecki K., Hallett M. (2012). Functional (psychogenic) movement disorders:. Curr. Opin. Neurol..

[b0195] de Lange F., Roelofs K., Toni I. (2007). Increased self-monitoring during imagined movements in conversion paralysis. Neuropsychologia.

[b0095] Diez I., Ortiz-Terán L., Williams B., Jalilianhasanpour R., Ospina J.P., Dickerson B.C., Keshavan M.S., LaFrance Jr W.C., Sepulcre J., Perez D.L. (2019). Corticolimbic fast-tracking: enhanced multimodal integration in functional neurological disorder. J. Neurol. Neurosurg. Psychiatry.

[b0100] Dreissen Y.E.M., Dijk J.M., Gelauff J.M., Zoons E., van Poppelen D., Contarino M.F., Zutt R., Post B., Munts A.G., Speelman J.D., Cath D.C., de Haan R.J., Koelman J.HTM., Tijssen M.A.J. (2019). Botulinum neurotoxin treatment in jerky and tremulous functional movement disorders: a double-blind, randomised placebo-controlled trial with an open-label extension. J. Neurol. Neurosurg. Psychiatry.

[b0105] Du Y., Allen E.A., He H., Sui J., Wu L., Calhoun V.D. (2016). Artifact removal in the context of group ICA: a comparison of single-subject and group approaches: artifact removal in the context of group ICA. Hum. Brain Mapp..

[b0110] Du Y., Fan Y. (2013). Group information guided ICA for fMRI data analysis. NeuroImage.

[b0115] Du, Y., Fu, Z., Sui, J., Gao, S., Xing, Y., Lin, D., et al., 2019. NeuroMark: an adaptive independent component analysis framework for estimating reproducible and comparable fMRI biomarkers among brain disorders. medRxiv, 19008631.

[b0120] Edwards M.J., Adams R.A., Brown H., Parees I., Friston K.J. (2012). A Bayesian account of 'hysteria'. Brain.

[b0125] Edwards M.J., Bhatia K.P. (2012). Functional (psychogenic) movement disorders: merging mind and brain. Lancet Neurol..

[b0130] Edwards M.J., Fotopoulou A., Pareés I. (2013). Neurobiology of functional (psychogenic) movement disorders:. Curr. Opinion Neurol..

[b0135] Espay A.J., Ries S., Maloney T., Vannest J., Neefus E., Dwivedi A.K., Allendorfer J.B., Wulsin L.R., LaFrance W.C., Lang A.E., Szaflarski J.P. (2019). Clinical and neural responses to cognitive behavioral therapy for functional tremor. Neurology.

[b0140] Espinoza F.A., Vergara V.M., Damaraju E., Henke K.G., Faghiri A., Turner J.A., Belger A.A., Ford J.M., McEwen S.C., Mathalon D.H., Mueller B.A., Potkin S.G., Preda A., Vaidya J.G., van Erp T.G.M., Calhoun V.D. (2019). Characterizing whole brain temporal variation of functional connectivity via zero and first order derivatives of sliding window correlations. Front. Neurosci..

[b0145] Fink P., Hansen M.S., Søndergaard L. (2005). Somatoform disorders among first-time referrals to a neurology service. Psychosomatics.

[b0150] Friston K., Kilner J., Harrison L. (2006). A free energy principle for the brain. J. Physiol.-Paris.

[b0155] Haggard P. (2017). Sense of agency in the human brain. Nat. Rev. Neurosci..

[b0160] Hallett M. (2006). Psychogenic movement disorders: a crisis for neurology. Curr. Neurol. Neurosci. Rep..

[b0165] Hassa T., de Jel E., Tuescher O., Schmidt R., Schoenfeld M.A. (2016). Functional networks of motor inhibition in conversion disorder patients and feigning subjects. NeuroImage: Clinical.

[b0170] Hentschke, H., Stüttgen, M.C., 2011. Computation of measures of effect size for neuroscience data sets. Eur. J. Neurosci. 34, 1887–1894. 10.1111/j.1460-9568.2011.07902.x 22082031

[b0175] van der Horn H.J., Vergara V.M., Espinoza F.A., Calhoun V.D., Mayer A.R., Naalt J. (2019). Functional outcome is tied to dynamic brain states after mild to moderate traumatic brain injury. Hum. Brain. Mapp..

[b0180] Hutchison R.M., Womelsdorf T., Allen E.A., Bandettini P.A., Calhoun V.D., Corbetta M., Della Penna S., Duyn J.H., Glover G.H., Gonzalez-Castillo J., Handwerker D.A., Keilholz S., Kiviniemi V., Leopold D.A., de Pasquale F., Sporns O., Walter M., Chang C. (2013). Dynamic functional connectivity: promise, issues, and interpretations. NeuroImage.

[b0185] de Jong B.M. (2019). Free will emerges from a multistage process of target assignment and body-scheme recruitment for free effector selection. Front. Psychol..

[b0190] Kaiser R.H., Whitfield-Gabrieli S., Dillon D.G., Goer F., Beltzer M., Minkel J., Smoski M., Dichter G., Pizzagalli D.A. (2016). Dynamic resting-state functional connectivity in major depression. Neuropsychopharmacol.

[b0200] Lui S.u., Li T., Deng W., Jiang L., Wu Q., Tang H., Yue Q., Huang X., Chan R.C., Collier D.A., Meda S.A., Pearlson G., Mechelli A., Sweeney J.A., Gong Q. (2010). Short-term Effects of Antipsychotic Treatment on Cerebral Function in Drug-Naive First-Episode Schizophrenia Revealed by “Resting State” Functional Magnetic Resonance Imaging. Arch. Gen. Psychiatry.

[b0205] Lurie D.J., Kessler D., Bassett D.S., Betzel R.F., Breakspear M., Kheilholz S., Kucyi A., Liégeois R., Lindquist M.A., McIntosh A.R., Poldrack R.A., Shine J.M., Thompson W.H., Bielczyk N.Z., Douw L., Kraft D., Miller R.L., Muthuraman M., Pasquini L., Razi A., Vidaurre D., Xie H., Calhoun V.D. (2020). Questions and controversies in the study of time-varying functional connectivity in resting fMRI. Network Neurosci..

[b0210] Mason M.F., Norton M.I., Van Horn J.D., Wegner D.M., Grafton S.T., Macrae C.N. (2007). Wandering minds: the default network and stimulus-independent thought. Science.

[b0215] Maurer C.W., LaFaver K., Ameli R., Epstein S.A., Hallett M., Horovitz S.G. (2016). Impaired self-agency in functional movement disorders. Neurology.

[b0220] Nahab F.B., Kundu P., Gallea C., Kakareka J., Pursley R., Pohida T., Miletta N., Friedman J., Hallett M. (2011). The neural processes underlying self-agency. Cereb. Cortex.

[b0225] Nicholson T.R., Carson A., Edwards M.J., Goldstein L.H., Hallett M., Mildon B., Nielsen G., Nicholson C., Perez D.L., Pick S., Stone J., Anderson D., Asadi-Pooya A., Aybek S., Baslet G., Bloem B.R., Brown R.J., Chalder T., Damianova M., David A.S., Epstein S., Espay A.J., Garcin B., Jankovic J., Joyce E., Kanaan R.A., Kozlowska K., LaFaver K., LaFrance W.C., Lang A.E., Lehn A., Lidstone S., Maurer C., Morgante F., Myers L., Reuber M., Rommelfanger K., Schwingenshuh P., Serranova T., Shotbolt P., Stebbins G., Tijssen M.A.J., Tinazzi M. (2020). Outcome measures for functional neurological disorder: a review of the theoretical complexities. JNP.

[b0230] Northoff G., Heinzel A., de Greck M., Bermpohl F., Dobrowolny H., Panksepp J. (2006). Self-referential processing in our brain—a meta-analysis of imaging studies on the self. NeuroImage.

[b0235] Potgieser A.R.E., de Jong B.M., Wagemakers M., Hoving E.W., Groen R.J.M. (2014). Insights from the supplementary motor area syndrome in balancing movement initiation and inhibition. Front. Hum. Neurosci..

[b0240] Power J.D., Barnes K.A., Snyder A.Z., Schlaggar B.L., Petersen S.E. (2012). Spurious but systematic correlations in functional connectivity MRI networks arise from subject motion. NeuroImage.

[b0245] Raichle M.E. (2015). The Brain's default mode network. Annu. Rev. Neurosci..

[b0250] Reid S., Wessely S., Crayford T., Hotopf M. (2002). Frequent attenders with medically unexplained symptoms: service use and costs in secondary care. Br. J. Psychiatry.

[b0255] Sepulcre J., Sabuncu M.R., Yeo T.B., Liu H., Johnson K.A. (2012). Stepwise connectivity of the modal cortex reveals the multimodal organization of the human brain. J. Neurosci..

[b0260] Stone J., Carson A., Duncan R., Roberts R., Warlow C., Hibberd C., Coleman R., Cull R., Murray G., Pelosi A., Cavanagh J., Matthews K., Goldbeck R., Smyth R., Walker J., Sharpe M. (2010). Who is referred to neurology clinics?—the diagnoses made in 3781 new patients. Clin. Neurol. Neurosurg..

[b0265] van der Stouwe A.M.M., Elting J.W., van der Hoeven J.H., van Laar T., Leenders K.L., Maurits N.M., Tijssen M.A.J. (2016). How typical are ‘typical’ tremor characteristics? Sensitivity and specificity of five tremor phenomena. Parkinsonism Related Disorders.

[b0270] Straka H., Simmers J., Chagnaud B.P. (2018). A new perspective on predictive motor signaling. Curr. Biol..

[b0275] Vergara V.M., Abrol A., Calhoun V.D. (2019). An average sliding window correlation method for dynamic functional connectivity. Hum. Brain Mapp..

[b0280] Vergara, V.M., van der Horn, H.J., Mayer, A.R., Espinoza, F.A., van der Naalt, J., Calhoun, V.D., 2019. Mild Traumatic Brain Injury Disrupts Functional Dynamic Attractors of Healthy Mental States. medRxiv, 19007906.

[b0285] Vergara V.M., Mayer A.R., Damaraju E., Calhoun V.D. (2017). The effect of preprocessing in dynamic functional network connectivity used to classify mild traumatic brain injury. Brain. Behav..

[b0290] Vergara V.M., Mayer A.R., Damaraju E., Hutchison K., Calhoun V.D. (2017). The effect of preprocessing pipelines in subject classification and detection of abnormal resting state functional network connectivity using group ICA. NeuroImage.

[b0295] Vergara V.M., Salman M., Abrol A., Espinoza F.A., Calhoun V.D. (2020). Determining the number of states in dynamic functional connectivity using cluster validity indexes. J. Neurosci. Methods.

[b0300] Voon V., Gallea C., Hattori N., Bruno M., Ekanayake V., Hallett M. (2010). The involuntary nature of conversion disorder. Neurology.

[b0305] Vuilleumier P. (2014). Brain circuits implicated in psychogenic paralysis in conversion disorders and hypnosis. Neurophysiologie Clinique/Clinical Neurophysiol..

[b0310] Xia, M., Wang, J., He, Y., 2013. BrainNet Viewer: a network visualization tool for human brain connectomics. PLoS One 8, e68910. 10.1371/journal.pone.0068910 PMC3701683 23861951

