# Supplementary figures and images for: The chronnectome as a model for Charcot’s ‘dynamic lesion’ in functional movement disorders
Source: Neuroimage Clin. 2020 Aug 13;28:102381. doi: 10.1016/j.nicl.2020.102381 (PMC7495110; doi:10.1016/j.nicl.2020.102381)

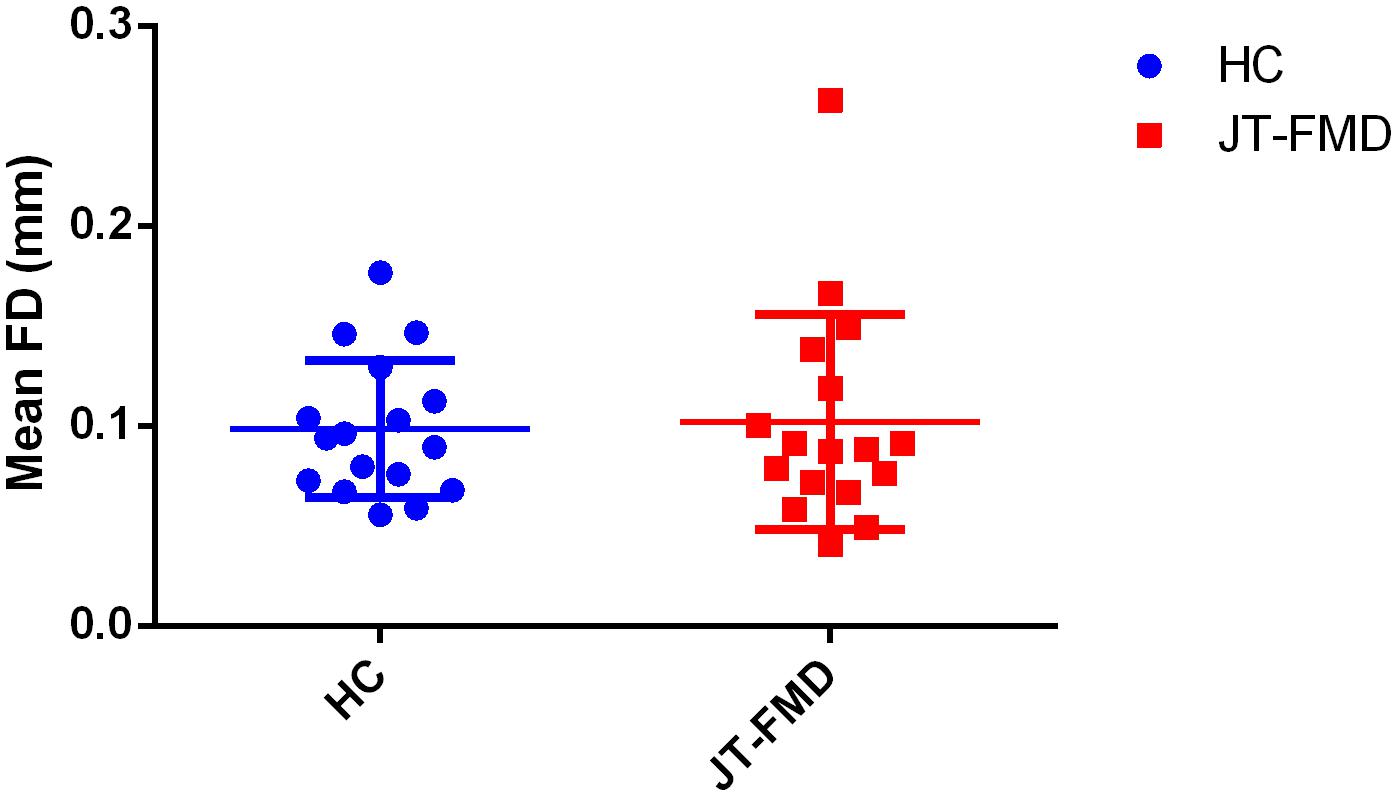

Supplement: Supplementary figure 1 — Mean framewise displacement for JT-FMD and HC . Lines reflect group mean ± standard deviation. There were no differences between groups (t=0.2294; p=0.82), which means that any movements present by extremities did not translate into head motion. [file fx1.jpg]

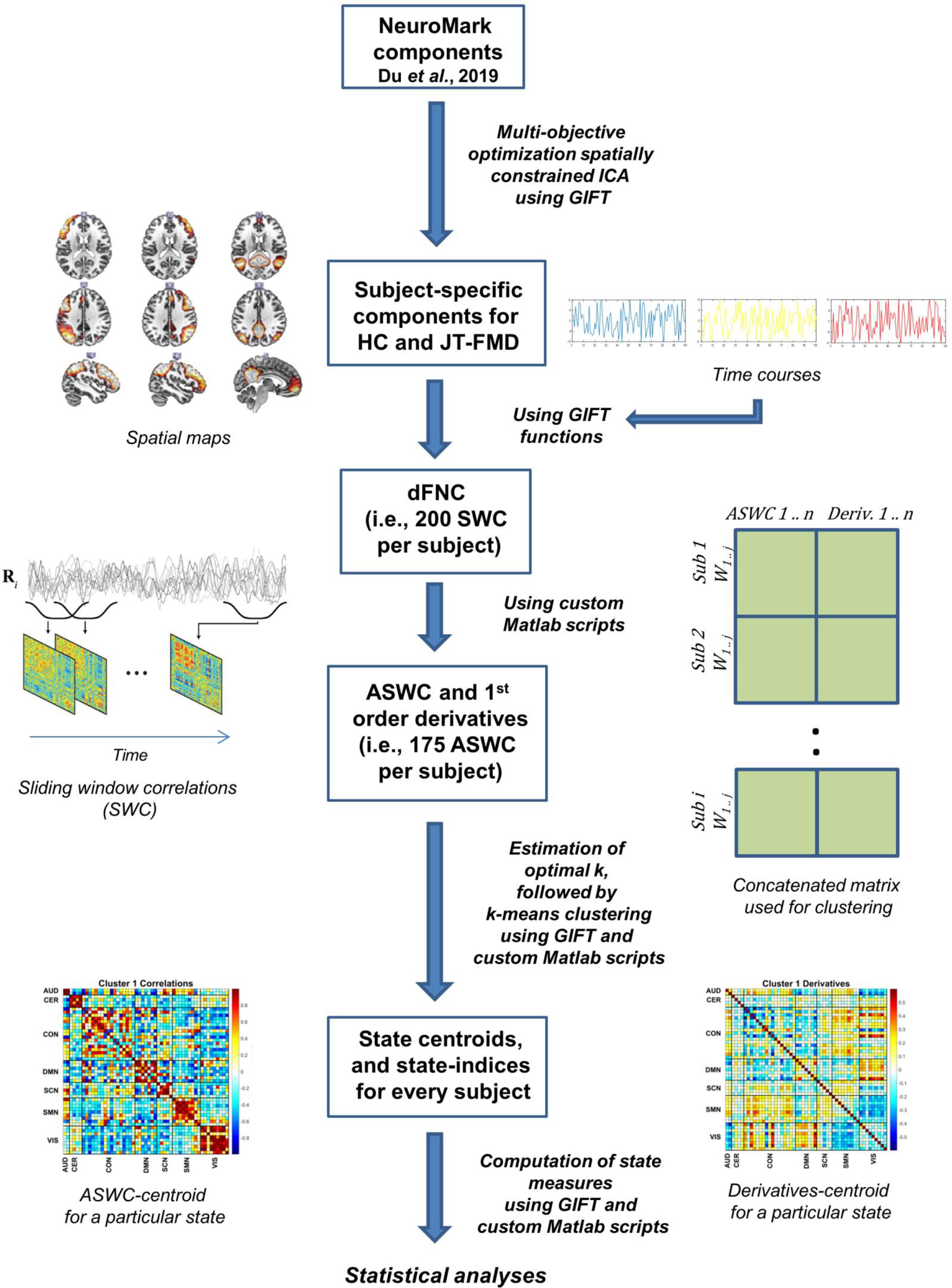

Supplement: Supplementary figure 2 — Dynamic functional network analytical pipeline [file fx2.jpg]

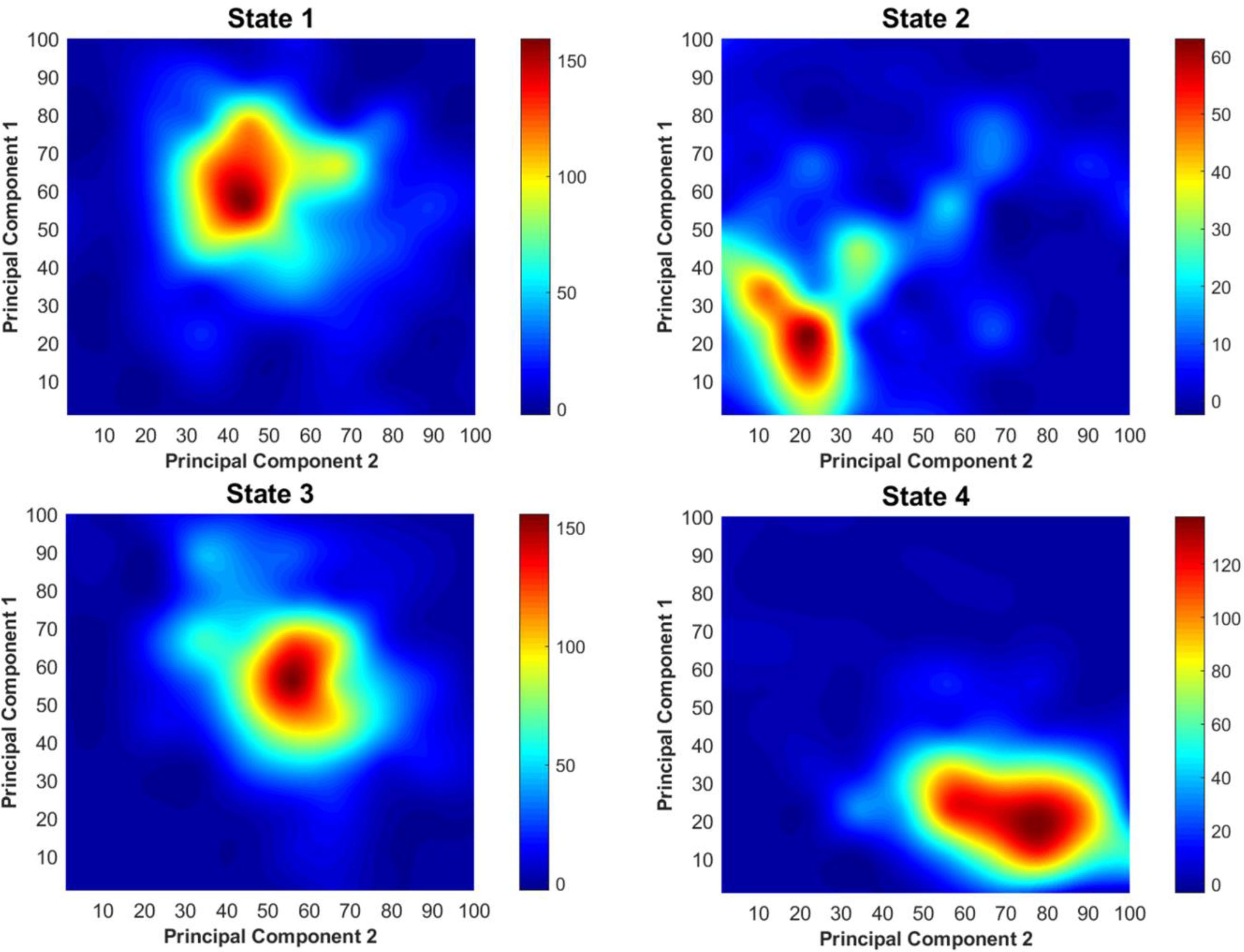

Supplement: Supplementary figure 3 — 2D representation of the four dynamic brain states extracted from this dataset. A principal component analysis was run on all high dimensional windowed data (i.e., 1378 ASWC + 1378 derivatives (variance scaled to ASWC)). The scores for the first two principal components (PC) were then smoothed, interpolated, and visualized in a bivariate histogram plot for each state separately using the membership indices obtained from k-means clustering (all steps were conducted using Matlab). Distinct density peaks, which represent the frequency of occurrence of a certain x (PC2) - y (PC1) score in the 2D histogram, can be appreciated for the different states, which support the structure of the clustered data. It can be noticed that density peaks of state 1 and 3 are relatively close to each other, which matches the observation that these states form an attractor in healthy subjects, as will be explained in more detail later in the manuscript. ASWC = average sliding window correlations. . [file fx3.jpg]

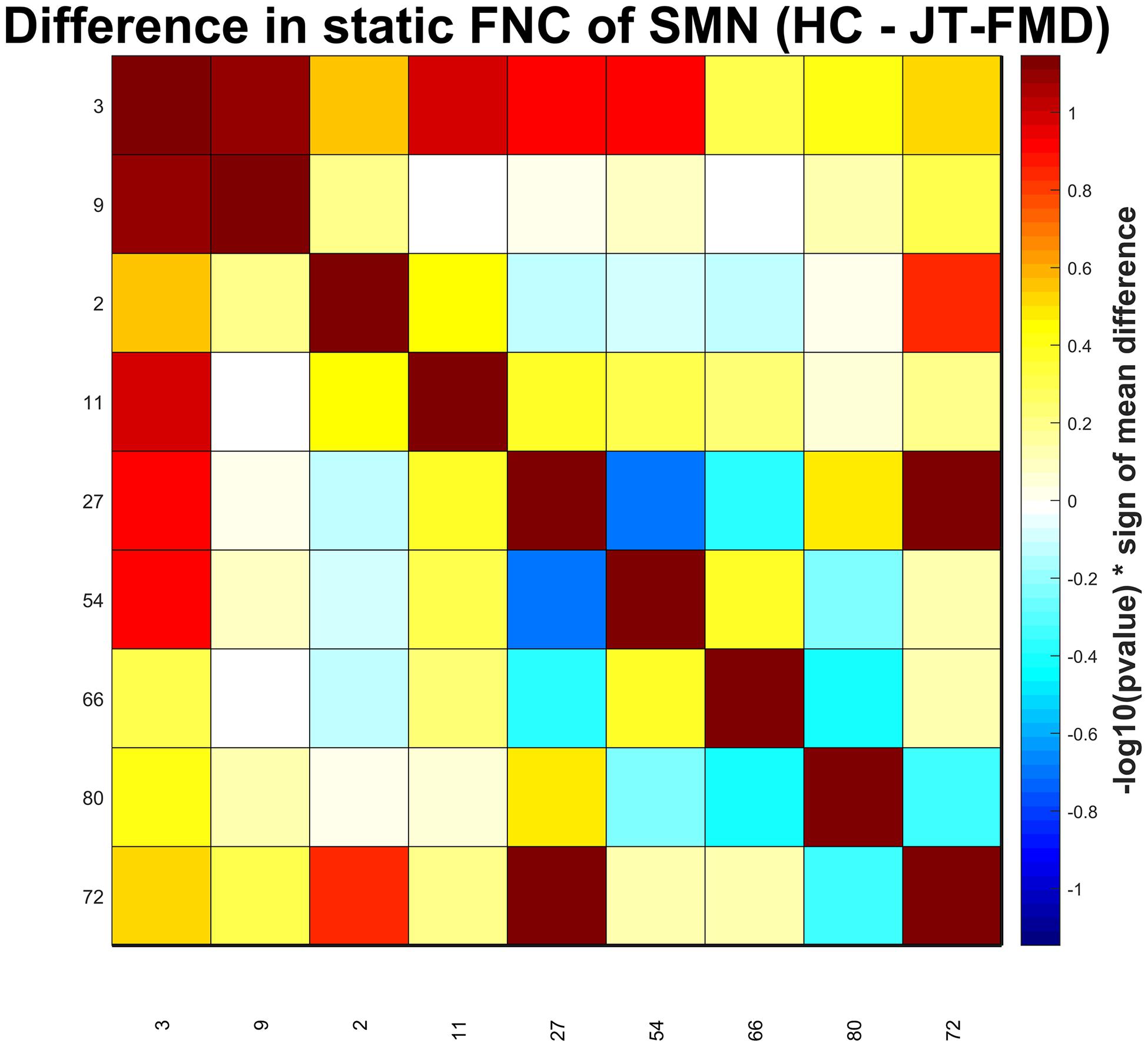

Supplement: Supplementary figure 4 — Static functional connectivity among the sensorimotor components between JT-FMD and HC. [file fx4.jpg]
